# Lumpectomy Combined with Adjuvant Radiotherapy Could Be a Treatment Option for Primary Squamous Cell Carcinoma of the Breast

**DOI:** 10.1155/2021/2497227

**Published:** 2021-12-02

**Authors:** Wei Xiang Qi, Lu Cao, Cheng Xu, Jiayi Chen

**Affiliations:** Department of Radiation Oncology, Ruijin Hospital, Shanghai Jiaotong University School of Medicine, Shanghai, China

## Abstract

**Background:**

To investigate the outcomes of primary squamous cell carcinoma (PSCC) of the breast undergoing radical surgery with or without adjuvant radiotherapy (RT).

**Materials and Methods:**

A population cohort with histologically diagnosed PSCC of the breast was identified from the SEER database. The Kaplan–Meier method and Cox-regression proportional hazards model was used to assess the impact of surgical types with or without adjuvant RT on the cause-specific survival (CSS) and overall survival (OS). A retrospective analysis of PSCC between Jan 2010 and Dec 2018 from our institute was performed.

**Results:**

A total of 515 patients with PSCC of the breast were included, 254 patients treated with mastectomy (MAST) alone, 78 with MAST + RT, 87 with lumpectomy (LUMP) alone, and 96 with LUMP + RT. The median follow-up time was 118 months (range: 0–379 months). In the multivariate Cox analyses, LUMP + adjuvant RT was an independent prognostic factor for CSS (*p* = 0.028) and OS (*p* = 0.048). Patients treated with LUMP + RT had better survival rates than patients who underwent lumpectomy (CSS, *p* = 0.034; OS, *p* = 0.0004), MAST alone (CSS, *p* = 0.0001; OS, *p* < 0.0001), and MAST + RT (CSS, *p* = 0.0001; OS, *p* = 0.0078), while postmastectomy RT did not significantly improve OS (*p* = 0.062) and CSS (*p* = 0.67) when compared to MAST alone. In addition, a total of 28 patients with PSCC of the breast were identified from our institute. All of these patients presented with estrogen receptor-negative type, and three of them had HER-2-positive PSCC; the median tumor size was 3 cm (range: 0.5–15 cm). Eight patients were treated with LUMP + adjuvant RT, thirteen with MAST, and seven with MAST + RT. Until the last follow-up of Sep 2021, 26 patients with PSCC were still alive and free of breast cancer, excepting that one patient treated with MAST and one patient with MAST + RT died from breast cancer.

**Conclusion:**

PSCC of the breast after radical surgery has a poor prognosis. Adjuvant RT after LUMP significantly improves survival of patients with PSCC of the breast. Further studies are still needed to investigate the role of adjuvant RT in PSCC of the breast after mastectomy.

## 1. Introduction

Primary squamous cell carcinoma (PSCC) of the breast is an epithelial type of metaplastic carcinoma according to the World Health Organization (WHO) classification of breast cancer [1]. The diagnosis of PSCC of the breast is established when more than 90% of malignant cells are of keratinizing squamous type excluding extramammary primary squamous cell carcinoma [2]. PSCC of the breast is a rare disease accounting for an estimated amount of less than 0.1% of all breast malignancies [3, 4]. As a result, most published studies are isolated case reports or case series from a single institute [3, 5–9].

Due to lack of adequate and accurate data, appropriate locoregional management, including surgical types or adjuvant radiotherapy (RT), for PSCC remains controversial, although most published studies have suggested that PSCC of the breast should be managed as invasive ductal carcinoma. As most patients present with large tumors, mastectomy (MAST) has been the most common surgical procedures for PSCC of the breast, although no data exist excluding breast-conserving surgery as an option [2, 10]. In addition, the clinical benefit of adjuvant RT among PSCC of the breast remains unresolved. Therefore, we perform the present study to investigate the clinical outcomes according to types of the surgical procedure and further assess the benefit of adjuvant RT for PSCC of the breast by using a population-based national registration database (Surveillance, Epidemiology, and End Results, SEER).

## 2. Materials and Methods

### 2.1. Ethical Statement

The present analysis is performed based on the SEER research database. The duration of the study was set from 1973 to December 2016, which was the record cutoff of the database. We obtained permission to access the data files from the SEER program by NCI with the reference number 11564-Nov 2019. As patient data identified from the database were deidentified and available to the public for research purposes, the ethical approval of the present study was waived by the local ethics committee.

In addition, a retrospective analysis of SCC of the breast cancer between 2010 and 2018 was performed in Ruijin Hospital Affiliated to Shanghai Jiaotong University School of Medicine, and a total of 28 cases were identified.

### 2.2. Treatment

Among the 28 included patients, eight patients were treated with LUMP + adjuvant RT, 13 with MAST, and 7 with MAST + RT. Fifteen patients underwent axillary lymph node dissection, and seven patients underwent sentinel lymph node biopsy (sentinel lymph node negative). Axillary lymph node dissection was not performed on two patients. Chemotherapy was administered to twenty-five patients, and adjuvant radiotherapy was administered to fifteen patients. Hormonal therapy was administered to one patient presented with low PR positivity (2%).

### 2.3. Search Strategy and Patient Cohort

All cases assigned the ICD-0–3 histology coded as 8070/3, 8071/3, 8072/3, 8073/3, 8074/3, 8075/3, 8076/3, 8077/3, and 8078/3 for primary tumor site of the breast were included in the present analysis. Age, race, sex, surgery, tumor size, TNM stage, number of positive LN, chemotherapy, radiotherapy, and sequence, grade, ER status, PR status, and year of diagnosis were abstracted from the SEER database. All the patients were categorized by the 6th edition of the American Joint Committee on Cancer/Union for International Cancer Control (AJCC/UICC) tumor-node-metastasis (TNM) staging system. Age was also converted to a binary data point using a cutoff of 50 years (≤ 50 vs. > 50 years). Race was classified as white or nonwhite. Surgery was classified as mastectomy (MAST) or lumpectomy (LUMP). Those with unknown surgery or no surgery status were excluded from analysis. Adjuvant radiotherapy was classified into yes or no. Therefore, locoregional treatment regimens were then divided into the following four groups: LUMP, LUMP + RT, MAST, and MAST + RT. The primary tumor site of the breast was determined via the ICD-0–3 site code. Adjuvant chemotherapy was classified into yes or no/unknown. Data for some analyzed variables (tumor stage, hormonal receptor status, tumor size, and number of positive LN) were incomplete. Those without data were excluded from statistical analysis involving those variables. We extracted the data using case listing session of SEER *∗* Stat 8.3.5 software.

### 2.4. Statistical Methods

In the SEER database, survival is calculated as the number of months from cancer diagnosis to the date of death. For the estimation of cancer-specific survival (CSS), women who died from causes other than PSCC of the breast were censored. The 10-year CSS and OS were calculated using the Kaplan–Meier method. Baseline clinicopathological characteristics were compared between the treatment groups by using chi-square and Fisher's exact probability tests. Baseline clinical variables were included in the univariate and multivariate survival analyses. The variables which had *p* < 0.1 in univariate analysis were put into the multivariate analysis. The statistical analysis was performed using the software of NCSS 11 Statistical Software (2016) (NCSS, LLC, Kaysville, Utah, USA, ncss.com/software/ncss). The statistical difference was considered as significant when *p* < 0.05.

## 3. Results

### 3.1. Baseline Characteristics

Between 1973 and 2016, a total of 515 patients with PSCC of the breast were identified from the SEER database. In the entire cohort, all of the patients were female and treated with radical surgery. Approximately 83.9% of them were white, and 16.1% were nonwhite. The median age of the PSCC of breast was 66 years, with 81.8% of the patients older than 50 years. The median tumor size was 3.55 cm (range: 0.2–52 cm). As for the local treatment strategies for PSCC of the breast, 254 patients (49.3%) were treated with mastectomy (MAST) alone, 78 (15.1%) with MAST + RT, 87 (16.9%) with lumpectomy (LUMP) alone, and 96 (18.6) with LUMP + RT. Approximately, 41.7% of them received adjuvant chemotherapy. In consistent with previous reports, the expression of hormonal receptor in PSCC of the breast was relatively low and 19.0% (71/374) and 11.3% (42/327) of patients were positive for ER and PR, respectively. In addition, half of the included patients (56.6%) presented with stage II (243/429), followed by stage I (22.1%) and stage III (21.2%). The baseline characteristics are listed in Table 1.

### 3.2. Survival Analysis

**Figure 1:**
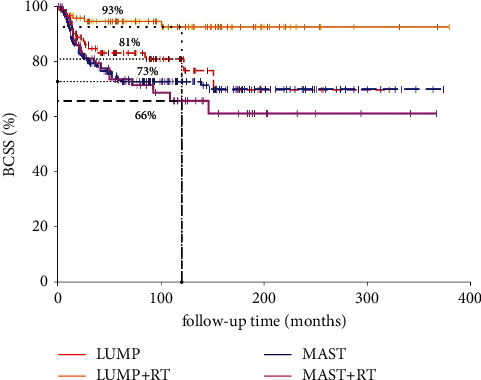
Kaplan–Meier analysis for BCSS of PSCC of the breast according to treatment regimens.

**Figure 2:**
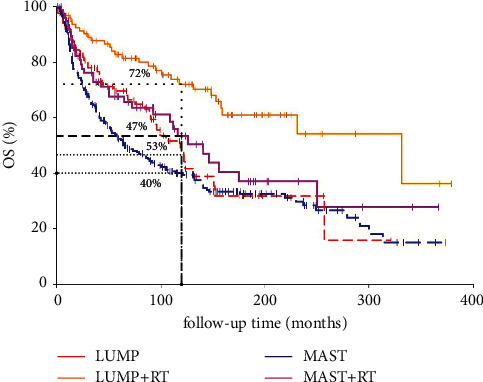
Kaplan–Meier analysis for OS of PSCC of the breast according to treatment regimens.

The median follow-up time was 118 months (range: 0–379 months). A total of 122 patients died of breast cancer. The 10-year CSS and OS were 77% and 49%, respectively. For the Kaplan–Meier analysis, the 10-year CSS was 93%, 81%, 73%, and 66% for patients treated with LUMP + RT, LUMP, MAST, and MAST + RT, respectively (*p* = 0.0006, Figure 1). As a result, an absolute CSS benefit of 12% at 10 years was seen in patients who were treated with LUMP + adjuvant RT compared to patients that received LUMP alone (*p* < 0.001). However, no significant CSS difference was observed between the MAST group and MAST + RT group (*p* = 0.67). In addition, the 10-year OS for patients treated with LUMP + RT, LUMP, MAST, and MAST + RT was 72%, 47%, 53%, and 40%, respectively (*p* < 0.001, Figure 2). When stratified by treatment regimens, LUMP + adjuvant RT significantly improved OS when compared to LUMP alone (*p* = 0.0034). In addition, adjuvant RT after mastectomy among PSCC patients had a tendency to improve OS (*p* = 0.06). Furthermore, it should be noted that the prognosis of PCSS who were treated with mastectomy ± adjuvant RT was poorer than that treated with LUMP ± adjuvant RT. One possible explanation for this finding was that the tumor characteristics of PSCC patients in the mastectomy arm are more aggressive; thus, the prognosis of this patient cohort in mastectomy will be slightly worse than that in the LUMP group. As shown in Table 1, more PSCC patients presented with stage III who were treated with mastectomy (18.5% in MAST alone (47/254) and 38.5% in MAST + RT (30/78) vs. 6.4% in LUMP (5/87) and 9.4% in LUMP + RT (9/96)). Therefore, further study is still needed to investigate the role of adjuvant postmastectomy radiotherapy in PSCC of the breast after mastectomy.

### 3.3. Univariate and Multivariate Analyses for CSS and OS

Data regarding age, race, TNM stage, ER status, PR status, grade, year of diagnosis, chemotherapy, and treatment regimens were included in univariate Cox-regression analysis (Table 2). Our results indicated that race (HR 0.64, *p* = 0.061), tumor stage (HR, 2.08, 4.19, and 9.02, *p* < 0.05), nodal status (HR 2.89 and 4.41, both *p* < 0.001), and treatment regimen (HR 0.29, *p* = 0.01) were significantly related to CSS of PSCC. Given the limitations of univariate analysis, multivariable Cox analysis was performed to investigate the independent factors associated with CSS (Table 3). Our findings showed that node positivity (HR 2.16, and 3.03, *p* = 0.01) was an independent predictor for worse CSS, while the prognosis of PSCC patients treated with LUMP + adjuvant RT was significantly better than that in patients treated with LUMP alone (HR 0.29, *p* = 0.028, Table 3). We also performed regression analysis to investigate the risk factors associated with OS. Univariate analysis indicated that age (HR 3.39, *p* < 0.001), stage (HR 3.90, *p* < 0.001), *T* stage (HR 2.30 and 4.31, *p* < 0.001), nodal status (HR 1.53 and 2.98, *p* < 0.05), PR status (HR 0.48, *p* = 0.015), and treatment regimens (HR 0.48, *p* = 0.0024) were significantly related to OS of PSCC. Multivariate analysis demonstrated that age, PR status, chemotherapy, *T* stage, nodal status, and treatment regimens were independent factors for OS among PSCC patients (Table 3).

### 3.4. Clinical and Pathological Data of Our Cohort

A total of 28 patients with PSCC of the breast were identified from our institute. The baseline characteristics of the included patients are listed in Table 4. The median age of included patients was 54.5 years (range: 39–80 years). All of these patients presented with hormonal receptor-negative type excepting for one patient with low PR positivity (2%) and three of them with HER-2-positive PSCC; the median tumor size was 3 cm (range: 0.5–15 cm). Twelve patients presented with pure PSCC, and sixteen patients with mixed PSCC. Among the mixed PSCC, most of them were PSCC mixed with invasive carcinoma of no special type (14 patients) and the remaining two patients were PSCC mixed with spindle cell carcinoma. Eight patients were treated with LUMP + adjuvant RT, 13 with MAST, and 7 with MAST + RT. Epirubicin (E) and cyclophosphamide (C) followed by docetaxel (T) (EC-T) was the most commonly used adjuvant chemotherapy regimen for PSCC of the breast. And, fifteen patients received adjuvant radiotherapy. Until the last follow-up of Sep 2021, 26 patients with PSCC were still alive and free of breast cancer, excepting that one patient treated with MAST and one patient treated with MAST + RT died from breast cancer.

## 4. Discussion

As reported in the literature, PSCC of the breast is an exceedingly rare malignancy. Although it was firstly reported more than 100 years ago [11], most published studies were care reports or small retrospective cohorts from a single institution [2, 5–8, 12, 13]. Therefore, the clinical characteristics and outcomes of PSCC of the breast are limited, although most researchers who achieved a relative consistent observation across many studies indicated that PSCC has aggressive histological features at presentation and has poor outcomes [14]. In a series of 31 patients with localized breast SCC reported by Hennessy et al. [15], the median relapse-free survival and overall survival was 20 months and 37 months, with a 5-year survival of 40%. Median survival from the time to recurrent disease was 14 months (range: 2–86 months). Subsequently, Yadav et al. [16] reported a larger series of 455 patients with PSCC of the breast based on the SEER database and found that PSCC was an aggressive tumor with a poor survival and the 1-year and 5-year cause-specific survival was 81.6% and 63.5%, respectively. Older age and higher tumor or nodal stages at presentation were independent predictors of poor survival for locoregional stages. However, until now, the optimal surgical procedure and the clinical value of adjuvant RT in patients with PSCC of the breast after surgery are still not clear, although mastectomy with axillary clearance is recommended as the initial treatment for PSCC of the breast by most physicians. As a result, we conduct the present study to investigate the clinical differences for surgical procedures and further analyze the effect of adjuvant RT on PSCC of the breast using a population-based national registration database.

A total of 515 patients with PSCC of the breast are finally included for analysis in the present study. In consistent with previous studies, most cases of PSCC of the breast occur in elderly patients (81.9% older than 50 years), with a median age of 66 years. In addition, PSCC of the breast has low ER or PR positivity (19.0% in ER positivity and 11.4% in PR positivity) and high histologic grade (59.5% in poor or undifferentiated). All of these features indicate that PSCC of the breast has a poor prognosis. Indeed, the 10-year OS of PSCC of the breast in the present study is 49%, which is significantly lower than the prognosis of invasive breast cancer (10-year survival is about 80%) [17]. Therefore, the initial treatment for PSCC of the breast is surgical procedure by mastectomy with axillary clearance. Breast conservative surgeries in these patients are not usually possible because most patients present with locally advanced disease. In our series, 332 patients (64.5%) treat with mastectomy and the remaining patients receive breast-conserving surgery. Radiotherapy, as an important adjuvant therapy, is widely used in invasive breast cancer patients after breast-conserving therapy or in patients with high-risk factors after mastectomy. Therefore, radiotherapy may be important in PSCC of the breast. Prior to the present study, in a series of 25 patients with PSCC of the breast who received adjuvant radiotherapy as a part of their primary treatment, Ogita et al. found that PSCC of the breast had a high incidence of locoregional recurrence, especially of in-field recurrence. In our study, a total of 168 patients (32.6%) receive adjuvant RT and adjuvant RT + BCS significantly improves CSS (*p* = 0.0034) and OS (*p* < 0.001) in comparison with BCS alone. We also find that adjuvant RT after mastectomy among PSCC patients has a tendency to improve OS (*p* = 0.06), but not for CSS (*p* = 0.67). Multivariate Cox analysis indicates that the treatment regimen is an independent predictor for OS and CSS. However, data on cancer recurrence could not be made available from the SEER database, and thus, we were unable to compare the locoregional recurrence rate in PSCC of the breast treated with or without radiotherapy. Based on our findings, adjuvant RT should be recommended for patients with PSCC of the breast after breast-conserving surgery. And, LUMP + RT could be an alternative treatment option for early-stage PSCC of the breast. But, the role of adjuvant RT after mastectomy is still needed to be investigated in further studies.

Inconsistent with findings from SEER data, 23 patients (82.1%) were older than 50 years with a median age of 54.5 years. In addition, all of these patients presented with hormonal receptor-negative type, excepting for one patient with low PR positivity (2%) and three of them with HER-2-positive PSCC. Two patients with HER-2-positive PSCC were treated with trastuzumab without disease recurrence. As for the survival outcomes of PSCC in our cohort, 26 patients with PSCC were still alive and free of breast cancer, excepting that one patient treated with MAST and one patient treated with MAST + RT died from breast cancer. Due to the limited sample size, whether LUMP + RT would be superior to MAST alone for PSCC of the breast remains undetermined in further studies.

So far, no adjuvant chemotherapy regimens for PSCC of the breast were established. Published studies indicated that PSCC of the breast was resistant to standard chemotherapy (cyclophosphamide, methotrexate, 5-FU, and adriamycin) for invasive breast cancers. Therefore, some researchers proposed the use of a cisplatinum-based chemotherapeutic regimen, which is commonly used for squamous cell carcinoma in the other sites. In our study, we find that adjuvant chemotherapy is an independent predictor for OS, but not for BCSS. However, the specific chemotherapy regimens used could not be available from the SEER database. In our cohort data, 25 PSCC patients received adjuvant chemotherapy. Of them, fifteen patients (60%) were treated with epirubicin (E) and cyclophosphamide (C) followed by docetaxel (T) (EC-T), four patients were treated with carboplatin-based chemotherapeutic regimen, two patients were treated with cyclophosphamide and docetaxel, one patient was treated with epirubicin (E) and cyclophosphamide (C) followed by paclitaxel (p), one patient was treated with epirubicin, cyclophosphamide, and 5-FU, one patient was treated with capecitabine alone, and the remaining one patient with unknown chemotherapy regimen.

Our study still has several limitations. First of all, selection bias existed among the four groups. For example, more patients with stage III receives MAST alone (51.6%) or MAST + RT (33%), when compared to LUMP alone (5.5%) and LUMP + RT (9.9%). Due to the rarity of this disease, we could not perform the PSM method to decrease the bias resulting from unevenly distributed measured covariates. Secondly, there is a sizeable amount of missing data regarding tumor stage and hormonal receptor status in the SEER database, which could be another source of bias. Thirdly, according to NCCN guideline, omitting radiotherapy in SCC patients undergoing lumpectomy remains unknown, especially for those with poor histology and who are young age. In our study, a total of 87 SCC patients were treated with LUMP alone. Of them, 17 patients were younger than 50 years. In addition, whether adjuvant radiotherapy should be performed for patients with stages I and II undergoing mastectomy remains undetermined, especially for those with negative lymph metastasis. Both of them would impact disease recurrence and survival. Finally, disease recurrence is not obtained from the SEER database. Therefore, we are unable to determine recurrence patterns and investigate factors associated with disease-free survival and locoregional relapse-free survival.

## 5. Conclusion

In conclusion, our findings show that the prognosis of PSCC after radical surgery remains poor. Adjuvant RT after BCS significantly improves survival when compared to BCS alone. Further studies are still needed to investigate the role of adjuvant RT in PSCC after mastectomy.

## Figures and Tables

**Table 1:** Baseline characteristics according to treatment regimens.

Characteristics	*n*	MAST (%)	MAST + RT (%)	LUMP (%)	LUMP + RT (%)	*p*
*Year of study*						
1983–1993	56	38 (15.0%)	6 (7.7%)	7(8.0%)	5 (5.2%)	0.012
1994–2004	209	103 (40.6%)	28 (35.9%)	29 (33.3%)	49(51.0%)	
2005–2016	250	113 (44.5%)	44 (56.4%)	51 (58.6%)	42 (43.8%)	
*Age* (*years*)						
≤50	93	31 (12.2%)	23 (29.5%)	17 (19.5%)	22 (22.9%)	0.002
>50	422	223 (87.8%)	55 (70.5%)	70 (80.5%)	74 (77.1%)	
*Race*						
Black	59	29 (11.4%)	8 (10.3%)	10 (11.5%)	12 (12.5%)	0.99
White	433	212 (83.5%)	67 (85.9%)	73 (83.9%)	81 (84.4%)	
Others	23	13 (5.1%)	3 (3.8%)	4 (4.6%)	3 (3.1%)	
*TNM stage*						
I	95	29 (11.4%)	2 (2.5%)	34 (39.1%)	30 (31.3%)	<0.001
II	243	131 (51.6%)	37 (47.4%)	32 (36.8%)	43 (44.8%)	
III	91	47 (18.5%)	30 (38.5%)	5 (5.7%)	9 (0.4%)	
Unknown	86	47 (18.5%)	9 (11.5%)	16 (18.4%)	14 (14.6%)	
*Tumor stage* (*n* = 429)						
T1	108	35 (13.8%)	4 (5.1%)	35 (40.2%)	34 (35.4%)	<0.001
T2	195	105 (41.3%)	27 (34.6%)	29 (33.3%)	34 (35.4%)	
T3	75	40 (15.7%)	20 (25.6%)	3 (3.4%)	12 (12.5%)	
T4	51	27 (10.6%)	18 (23.1%)	4 (4.6%)	2 (2.1%)	
Unknown	86	47 (18.5%)	9 (11.5%)	16 (18.3%)	14 (14.6%)	
*Nodal stage*						
N0	322	156 (61.4%)	39 (50%)	62 (71.3%)	65 (67.7%)	0.002
N1	72	32 (12.6%)	22 (28.2%)	6 (6.9%)	12 (12.5%)	
N2	21	11 (4.3%)	7 (9.0%)	1 (1.1%)	2 (2.1%)	
N3	10	6 (2.4%)	1 (1.3%)	0	3 (3.1%)	
Unknown	90	49 (19.3%)	9 (11.5%)	18 (20.7%)	14 (14.6%)	
*ER status*						
Positive	71	39 (15.4%)	11 (14.1%)	9 (10.3%)	12 (12.5%)	0.01
Negative	303	139 (54.7%)	53 (67.9%)	44 (50.6%)	67 (69.8%)	
Unknown	141	76 (29.9%)	14 (17.9%)	34 (39.1%)	17 (17.7%)	
*PR status*						
Positive	42	15 (5.9%)	11 (14.1%)	6 (6.9%)	10 (10.4%)	0.01
Negative	327	159 (62.6%)	53 (67.9%)	48 (55.2%)	67 (69.8%)	
Unknown	146	80 (31.5%)	14 (17.9%)	33 (37.9%)	19 (19.8%)	
*Grade*						
Well-differentiated	50	22 (33.3%)	7 (33.3%)	14 (41.2%)	7 (24.1%)	0.29
Moderately differentiated	122	66 (66.7%)	14 (66.7%)	20 (58.8%)	22 (75.9%)	
Poor or undifferentiated	253	117 (70.5%)	47 (82.5%)	40 (75.5%)	49 (73.1%)	
Unknown	90	49 (29.5%)	10 (17.5%)	13 (24.5%)	18 (26.9%)	
*Chemotherapy*						
Yes	206	86 (33.9%)	54 (69.2%)	17 (19.5%)	49 (51.0%)	<0.001
No/unknown	309	168 (66.1%)	24 (30.8%)	70 (80.5%)	47 (49.0%)	

MAST, mastectomy; LUMP, lumpectomy; RT, radiotherapy; ER, estrogen receptor; PR, progesterone receptor.

**Table 2:** Univariate analysis of breast cancer-specific survival and overall survival.

Characteristics	BCSS			OS		
	HR	95% CI	p	HR	95% CI	p
*Age* (*years*)						
≤50	1			1		
>50	1.38	0.81–2.36	0.23	3.39	2.16–5.31	<0.001
*Race*						
Others	1			1		
White	0.64	0.40–1.02	0.061	0.98	0.69–1.39	0.92
*Stage*						
I	1			1		
II	3.19	1.36–7.49	0.0079	1.47	1.01–2.16	0.046
III	11.63	4.93–27.44	<0.001	3.90	2.58–5.88	<0.001
*Tumor stage*						
T1	1			1		
T2	2.08	1.03–4.20	0.041	1.26	0.87–1.82	0.22
T3	4.19	1.98–8.45	0.0002	2.30	1.51–3.51	<0.001
T4	9.02	4.29–18.95	<0.001	4.31	2.78–6.68	<0.001
*Nodal status*						
N0	1			1		
N1	2.89	1.79–4.66	<0.001	1.53	1.07–2.19	0.019
N2-3	4.41	2.46–7.90	<0.001	2.98	1.94–4.57	<0.001
*ER status*						
Negative	1			1		
Positive	1.27	0.74–2.18	0.39	1.05	0.72–1.55	0.79
*PR status*						
Negative	1			1		
Positive	0.48	0.19–1.19	0.11	0.48	0.27–0.87	0.015
*Grade*						
Well-differentiated	1			1		
Moderately differentiated	1.08	0.48–2.42	0.85	0.97	0.61–1.53	0.89
Poor or undifferentiated	1.30	0.62–2.73	0.49	0.84	0.54–1.29	0.42
*Chemotherapy*						
No/unknown	1			1		
Yes	1.19	0.81–1.75	0.38	0.54	0.41–0.71	<0.001
*Treatment*						
LUMP only	1			1		
LUMP + RT	0.29	0.11–0.75	0.01	0.48	0.30–0.77	0.0023
MAST only	1.35	0.78–2.35	0.28	1.28	0.91–1.81	0.15
MAST + RT	1.51	0.79–2.90	0.21	0.91	0.58–1.43	0.67

MAST, mastectomy; LUMP, lumpectomy; RT, radiotherapy; ER, estrogen receptor; PR, progesterone receptor; BCSS, breast cancer-specific survival; OS, overall survival.

**Table 3:** Multivariate analysis of cause-specific survival and overall survival.

Characteristics	CSS			OS		
	HR	95% CI	*p*	HR	95% CI	*p*
*Race*						
Others	1			-		
White	0.88	0.51–1.50	0.63	-	-	-
*Age*						
≤50	-			1		
>50	-	-	-	1.92	1.03–3.56	0.039
*PR status*						
Negative	-			1		
Positive	-	-	-	0.45	0.24–0.83	0.011
*Chemotherapy*						
No/unknown	-			1		
Yes	-	-	-	0.45	0.31–0.66	<0.001
*Stage*						
I	1			1		
II	2.56	0.65–10.11	0.18	1.17	0.40–3.40	0.78
III	2.85	0.53–15.32	0.22	0.76	0.18–3.12	0.70
*Tumor stage*						
T1	1			1		
T2	0.83	0.28–2.45	0.74	1.20	0.46–3.10	0.71
T3	1.44	0.46–4.53	0.53	2.27	0.83–6.21	0.11
T4	2.65	0.68–10.34	0.16	5.94	1.63–21.70	0.0071
*Nodal status*						
N0	1			1		
N1	2.16	1.20–3.88	0.01	2.21	1.34–3.66	0.0019
N2-3	3.03	1.25–7.40	0.01	5.55	2.30–13.39	0.0001
*Treatment*						
LUMP only	1			1		
LUMP + RT	0.29	0.09–0.87	0.028	0.39	0.20–0.74	0.0048
MAST only	1.23	0.58–2.60	0.59	0.90	0.54–1.48	0.68
MAST + RT	0.79	0.32–1.90	0.59	0.57	0.30–1.10	0.093

MAST, mastectomy; LUMP, lumpectomy; RT, radiotherapy; ER, estrogen receptor; PR, progesterone receptor; BCSS, breast-cancer specific survival; OS, overall survival.

**Table 4:** Baseline characteristics of included PSCC in our institution.

Characteristics	Number
Age (median, range)	54.5 years (range: 39–80 years)
*ER status*	
Positive	0
Negative	28 (100%)
*PR status*	
Positive	1 (3.6%)
Negative	27 (96.4%)
*HER-*2 *status*	
Positive	3 (10.7%)
Negative	25 (89.3%)
*Ki-*67 *status*	
≥20%	26 (92.9%)
<20%	2 (7.1%)
Tumor size (median, range)	3 cm (range: 0.5–15 cm)
*Adjuvant chemotherapy*	
Yes	25 (89.3%)
No	3 (10.7%)
*Adjuvant radiotherapy*	
Yes	15 (53.6%)
No	13 (46.4%)
*Types of PSCC*	
Pure	12 (42.9)
Mixed	16 (57.1%)
*Tumor stage*	
T1	9 (32.1%)
T2	18 (64.3%)
T3	0
T4	1 (3.6%)
*Nodal status*	
N0	22 (78.6%)
N1	5 (17.9%)
N2	1 (3.6%)
*Treatment*	
LUMP + RT	8 (28.6%)
MAST only	13 (46.4%)
MAST + RT	7 (25%)

MAST, mastectomy; LUMP, lumpectomy; RT, radiotherapy; ER, estrogen receptor; PR, progesterone receptor.

## Data Availability

In the study, the authors obtained the data from the SEER database, which was open access for research purposes. The authors were permitted to access the data at the website (http://www.seer.cancer.gov) with the identifier, 11564-Nov 2019.
